# ﻿A new species of *Orchomenella* (Amphipoda, Tryphosidae) described from hydrothermal vent in the Okinawa Trough, Northwest Pacific

**DOI:** 10.3897/zookeys.1184.111420

**Published:** 2023-11-21

**Authors:** Yanrong Wang, Zhongli Sha, Xianqiu Ren

**Affiliations:** 1 Department of Marine Organism Taxonomy and Phylogeny, Institute of Oceanology, Chinese Academy of Sciences, Qingdao 266071, China; 2 Laoshan Laboratory, Qingdao 266237, China; 3 College of Biological Sciences, University of Chinese Academy of Sciences, Beijing 100049, China; 4 Shandong Province Key Laboratory of Experimental Marine Biology, Institute of Oceanology, Chinese Academy of Sciences, Qingdao, China

**Keywords:** COI, deep-sea fauna, morphology, *Orchomenellacompressa* sp. nov, taxonomy

## Abstract

A new species of the family Tryphosidae, *Orchomenellacompressa***sp. nov.**, is described from hydrothermal vents in the Okinawa Trough. This is the first known *Orchomenella* species found in vent fields. Important morphological characters that differentiate *O.compressa***sp. nov.** from its congeners are the absence of eyes, the compressed distal three articles of gnathopod 2, the shape of the posterior margin of epimerons 2 and 3, and the number of dorsal spines on the telson. The genetic divergence of the analyzed COI gene clearly supports this new taxon.

## ﻿Introduction

The family Tryphosidae was established as a subfamily in family Lysianassidae Dana, 1849 by Lowry and Stoddart (1997), separate from Lysianassinae based on following characters: molar of mandible asetose, apical margin of the outer plate of the maxilliped bearing robust setae, gnathopod 1 subchelate, and telson cleft. The genus *Orchomenella* Sars, 1890 contains 34 species (WoRMS 2023), which are found in various habitats ranging from shallow water to deep sea (Barnard 1969; De Broyer 1984, 1985; Jung et al. 2017). Molecular analysis indicates that *Orchomenella* is probably not monophyletic (Havermans et al. 2010), but the genus has been retained awaiting further evidence (Jung et al. 2017).

During a biodiversity survey of hydrothermal vents in the Okinawa Trough in the western Pacific conducted by the Chinese research vessel *Kexue* in 2014, several individuals referable to *Orchomenella* were collected. After careful examination, these specimens exhibited some distinctive characters differentiating them from known *Orchomenella* species. The present study describes this new species.

## ﻿Materials and methods

The present materials were collected by ROV Faxian suction sampler, together with *Iheyaspiralequios* Okutani, Sasaki & Tsuchida, 2000 and *Probathylepasfaxian* Ren & Sha, 2015, during expeditions to hydrothermal vents on the Okinawa Trough by the Institute of Oceanology, Chinese Academy of Sciences (IOCAS) in 2014. The specimens are deposited in the Marine Biological Museum, Chinese Academy of Sciences (MBMCAS), Qingdao, China. Specimens were examined and dissected using a dissecting microscope (Zeiss Discovery V20). Line drawings were made with a tablet (Wacom Intuos Pro PTH-851) and Adobe Photoshop CS6 (v. 13). Length measurement is made along the outline of the animal, beginning from the anterior margin of head to the end of the urosomite 3.

The COI sequence of *Orchomenellacompressa* sp. nov. (657 bp) was obtained from its mitochondrial genome by homologous alignment, and the mitochondrial genome of the new species was obtained by Illumina HiSeq sequencing. For Illumina pair-end sequencing of each strain, at least 3 μg of genomic DNA was used for sequencing library construction. Paired-end libraries with insert sizes of ~400 bp were prepared following Illumina’s standard genomic DNA library preparation procedure. Purified genomic DNA was sheared into smaller fragments with a desired size by Covaris, and blunt ends were generated by using T4 DNA polymerase. After adding an “A” base to the 3’ end of the blunt phosphorylated DNA fragments, adapters were ligated to the ends of the DNA fragments. The desired fragments were purified through gel-electrophoresis, then selectively enriched and amplified by PCR. The index tag was introduced into the adapter at the PCR stage, as appropriate, and we did a library quality test. Finally, the qualified Illumina pair-end library was used for Illumina NovaSeq 6000 sequencing (150 bp*2, Shanghai Biozeron Co., Ltd). The raw paired end reads were trimmed and quality controlled by Trimmomatic with parameters (SLIDINGWINDOW:4:15 MINLEN:75) (v. 0.36 http://www.usadellab.org/cms/uploads/supplementary/Trimmomatic). Clean data obtained by above quality control processes were used to do further analysis.

The obtained sequences were edited using Lasergene and aligned using MEGA v. 6 (Tamura et al. 2013). Except for *Orchomenellacompressa* sp. nov. sequenced in this study, the other 17 sequences from nine described species and the outgroup *Orchomenyx* (see Table 1 for GenBank accession numbers) were used for comparative and phylogenetic analyses. Maximum-likelihood (ML) analyses were performed online at W-IQ-TREE (Jana et al. 2016). Clade support was assessed with 1000 ML bootstrap replications. The genetic divergences between and within the 10 *Orchomenella* species were constructed in MEGA v. 6 (Tamura et al. 2013).

**Table 1.** Details of specimens and GenBank accession numbers used in this study.

Genus	Species	GenBank accession no.
*Orchomenella*	*O.compressa* sp. nov.	OR360510
	*O.pinguides*	HQ918313
	*O.franklini*	HQ918402
	*O.franklini*	HQ918406
	*O.gerulicorbis*	KP713918
	*O.pinguis*	FJ581801
	*O.minuta*	FJ581800
	*O.minuta*	FJ581799
	*O.cavimanus*	GU109264
	*O.rotundifrons*	MF124131
	*O.infinita*	MF124125
	*O.cavimanus*	HM054042
	*O.pinguides*	HM054031
	*O.rotundifrons*	MF124143
	*O.acanthura*	MH825758
	*O.acanthura*	MH825756
*Orchomenyx*	*O.schellenbergi*	HM054044
	*O.macronyx*	GU109231

## ﻿Systematics

**Order Amphipoda Latreille, 1816**

**Suborder Amphilochidea Boeck, 1871**

**Superfamily Lysianassoidea Dana, 1849**

**Family Tryphosidae Lowry & Stoddart, 1997**

**Genus *Orchomenella* Sars, 1890**

### ﻿ Orchomenella compressa sp. nov.

Taxon classificationAnimaliaAmphipodaTryphosidae

36129687-51E7-53D1-B53E-FC2330C3657B

https://zoobank.org/269C63F4-CFA2-45B1-9D5A-55CBFB4EED02

Figs 1 , 2 , 3 , 4

#### Materials examined.

***Holotype*.** MBM 286555, ♀ (6.2 mm), dissected, Okinawa Trough, 27°33'N, 126°58'E, RY0067, ROV-3, depth 1243 m, 16 April 2014. ***Paratype***: MBM 286555, ♂ (5.6 mm), same collection data as holotype.

#### Additional materials.

MBM 286566, 4♀♂, Okinawa Trough, 27°33'N, 126°58'E, RY0069, ROV-3, depth 1243 m, 16 Apr. 2014.

#### Description.

Body smooth; epimerons 1–3 smooth, posteroventral margin rounded; urosomite 1 bearing dorsal notch. Head: head deeper than long; lateral cephalic lobe large, subtriangular, subacute apically. Eyes: no trace visible in ethanol-preserved materials. Antenna 1 with peduncular article 1 longest, length ~1.5× width, without dorsodistal protrusion, article 3 subequal to article 2; flagellum 9-articulate, with callynophore, without calceoli; accessory flagellum long, 5-articulate, article 1 longest, not forming cap. Antenna 2 longer than antenna 1; peduncle without brush setae, article 4 longer than article 5; flagellum 7-articulate, shorter than peduncle, without calceoli.

***Mouthparts*.** Upper and lower lip typical for the genus. Mandible incisors symmetrical, smooth, subtriangular; left lacinia mobilis a long, slender peg; accessory setal row without distal setal tuft, both left and right with 3 short, slender, simple setae; molar well developed, columnar; palp 3-articulate, attached lower than molar, article 1 shortest, article 3 blade-like, shorter than article 2, fringed with long simple setae. Maxilla 1 with inner plate narrow with 2 pappose apical setae; outer plate with 11 setal-teeth in 7/4 crown arrangement; palp large, 2-articulate, with 9 terminal short robust setae and 1 long, plumose seta. Maxilla 2 inner plate narrower and slightly shorter than outer plate. Maxilliped with inner plate rectangular, distal margin with teeth and 1 or 2 robust setae; outer plate does not extend to distal margin of palp article 3, with 2 apical, robust setae; palp 4-articulate, dactylus nearly as long as article 3, unguis present.

**Figure 1.**
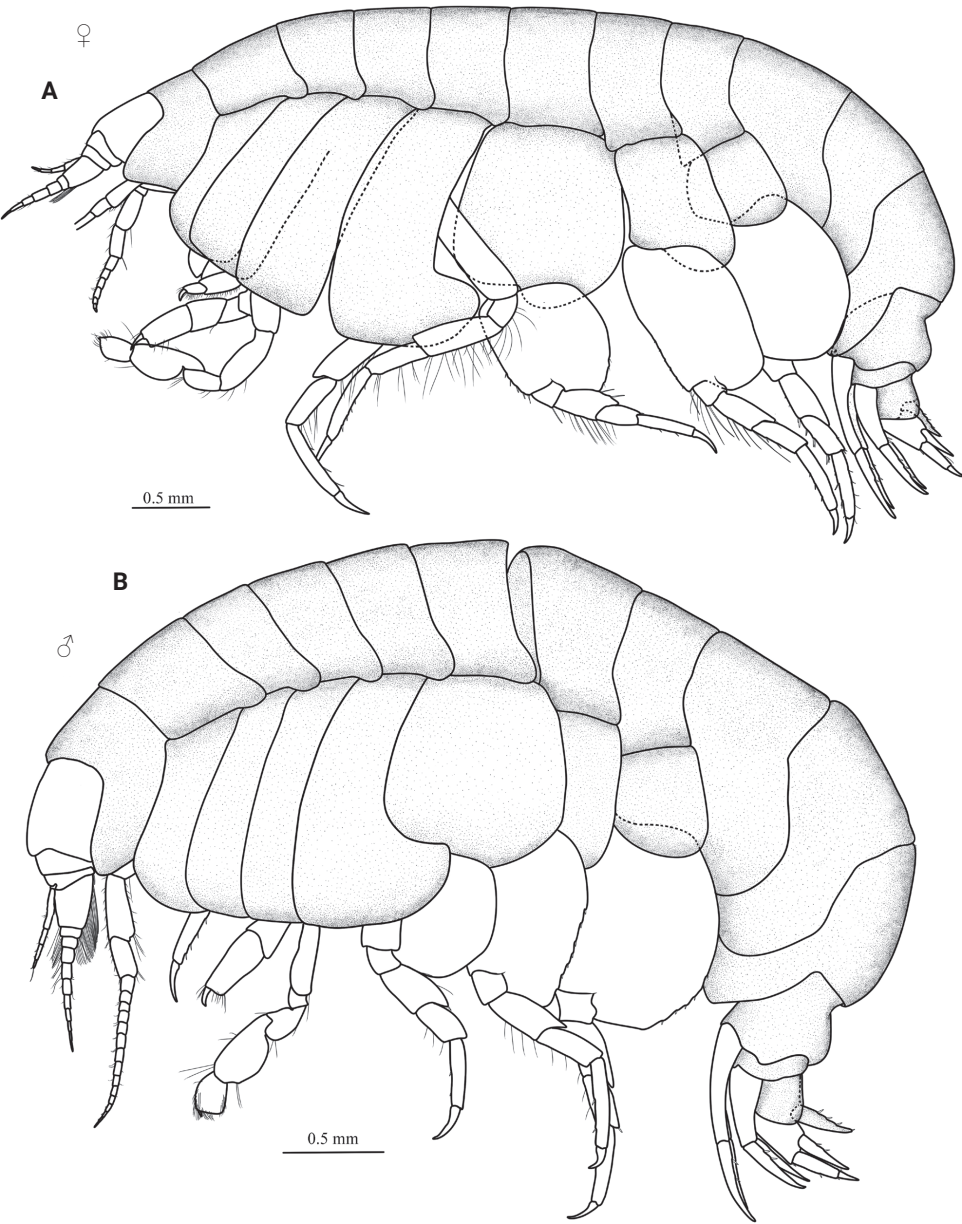
*Orchomenellacompressa* sp. nov., MBM 286555 **A** holotype, ♀ (6.2 mm) **B** paratype, ♂ (5.6 mm).

Coxae 1–4 longer than broad, overlapping. Gills present on coxae 2–7; pereopod 5 and 6 bearing accessory gill. Oostegites slender, present on coxae 2–5.

***Pereopods*.** Gnathopod 1 subchelate; coxa anterior margin slightly concave, posterior margin nearly straight; basis slender, anterior margin fringed with thin, simple setae; ischium shorter than merus; merus shorter than carpus, with distal margin bearing 4 long setae; carpus slightly shorter than propodus; propodus subrectangular, posterior margin straight, posterodistal corner with a group of simple setae, without robust setae; dactylus simple, as long as palm. Gnathopod 2 minutely chelate, with distal 3 articles distinctly compressed compared to other articles; coxa large, subequal in size to coxa 3; ischium 1.8× longer than merus; carpus compressed, 1.6× longer than propodus, posterior margin strongly convex, anterior margin nearly straight; propodus subrectangular; dactylus reaching corner of palm, tufts of setae covering most of distal part of propodus. Pereopod 3 coxa large; merus with posterior margin bearing simple, long setae; propodus with posterior margin bearing small, robust setae; dactylus slender. Pereopod 4 coxa large, with large posteroventral lobe, anterior margin convex; ischium to carpus with posterior margin bearing simple, long setae; propodus nearly as long as merus, posterior margin with small robust setae; dactylus slender. Pereopod 5 coxa slightly deeper than wide, posterior lobe slightly larger than anterior one; basis broadly expanded with posterior and anterior margin bearing small robust setae; merus longer than carpus, slightly expanded posteriorly; propodus with anterior margin bearing 4 small, robust setae; dactylus slender. Pereopod 6 coxa posterior lobate; basis expanded, posterior and anterior margin bearing small, robust setae; merus subequal in length to carpus, anterior margin with robust setae, posterior margin bearing 1 distal and 1 subdistal seta; propodus with small, robust setae along anterior and posterior margins; dactylus slender. Pereopod 7 with coxa small; basis wider than that of pereopod 6, posterior margin broadly rounded; distal 5 articles similar to that of pereopod 6.

**Figure 2.**
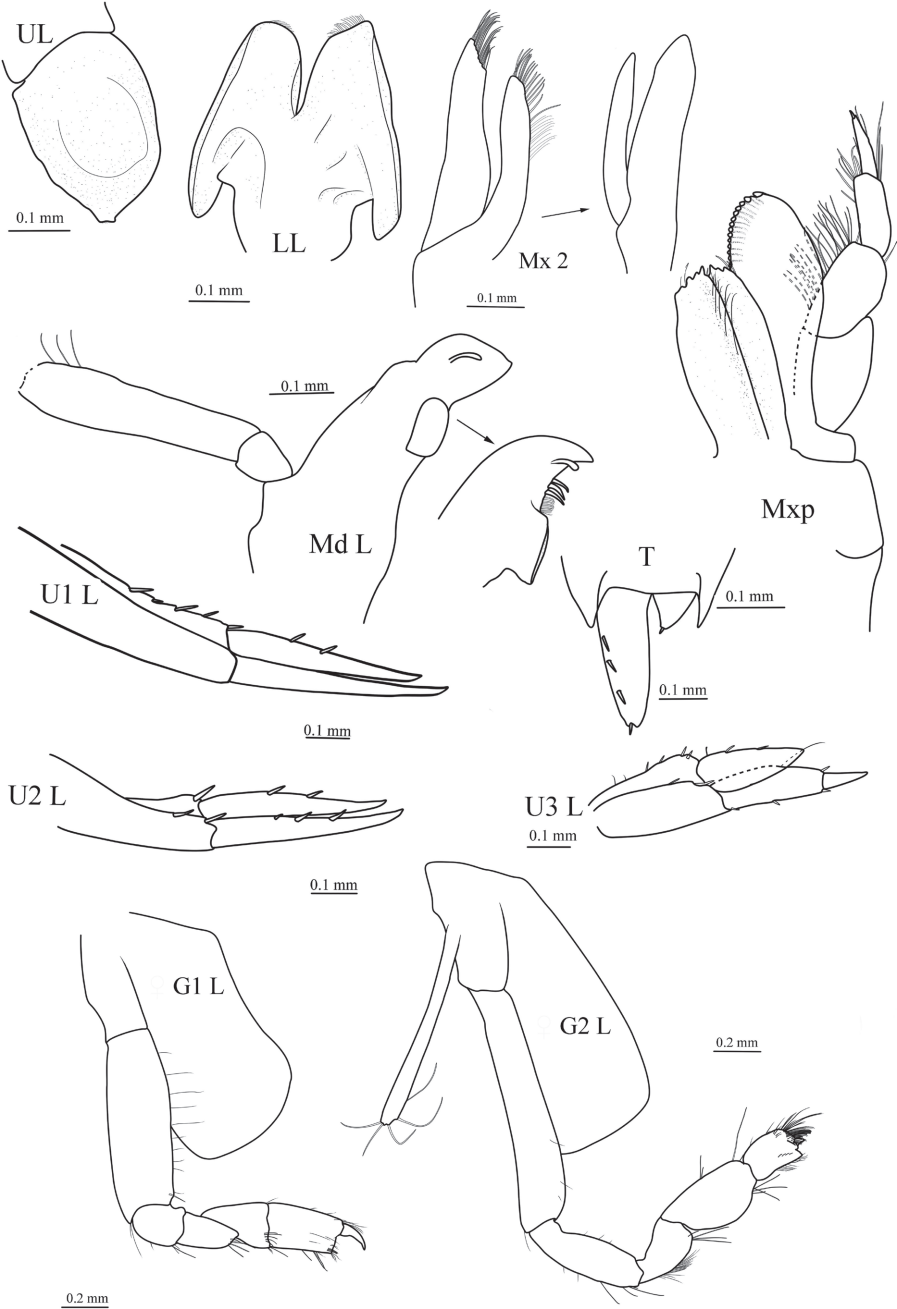
*Orchomenellacompressa* sp. nov., MBM 286555, holotype, ♀ (6.2 mm): UL, upper lip; LL, lower lip; Md L, left mandible; M×2, maxilla 2; Mxp, maxilliped; U1 L, left uropod 1; U2 L, left uropod 2; U3 L, left uropod 3; T, telson.

***Uropods and telson*.** Uropod 1 peduncle longer than rami, with 4 dorsomedial and 1 apicomedial robust setae; outer ramus slightly longer than inner ramus; inner ramus with 2 dorsal robust setae. Uropod 2 peduncle subequal in length to rami, with 1 apicolateral, 1 dorsolateral, and 1 apicomedial robust seta; outer ramus slightly longer than inner ramus, with 3 dorsal, robust setae; inner ramus with 2 dorsal, robust setae. Uropod 3 peduncle slightly shorter than outer ramus, with 6 robust setae; outer ramus 2-articulate, article 1 with 2 lateral and 2 distal robust setae, article 2 about 0.5× length of article 1; inner ramus not reaching to base of article 2 of outer ramus, with 2 lateral robust setae. Telson cleft, about 68%, bearing 3 dorsal and 1 apical robust setae on each side.

**Figure 3.**
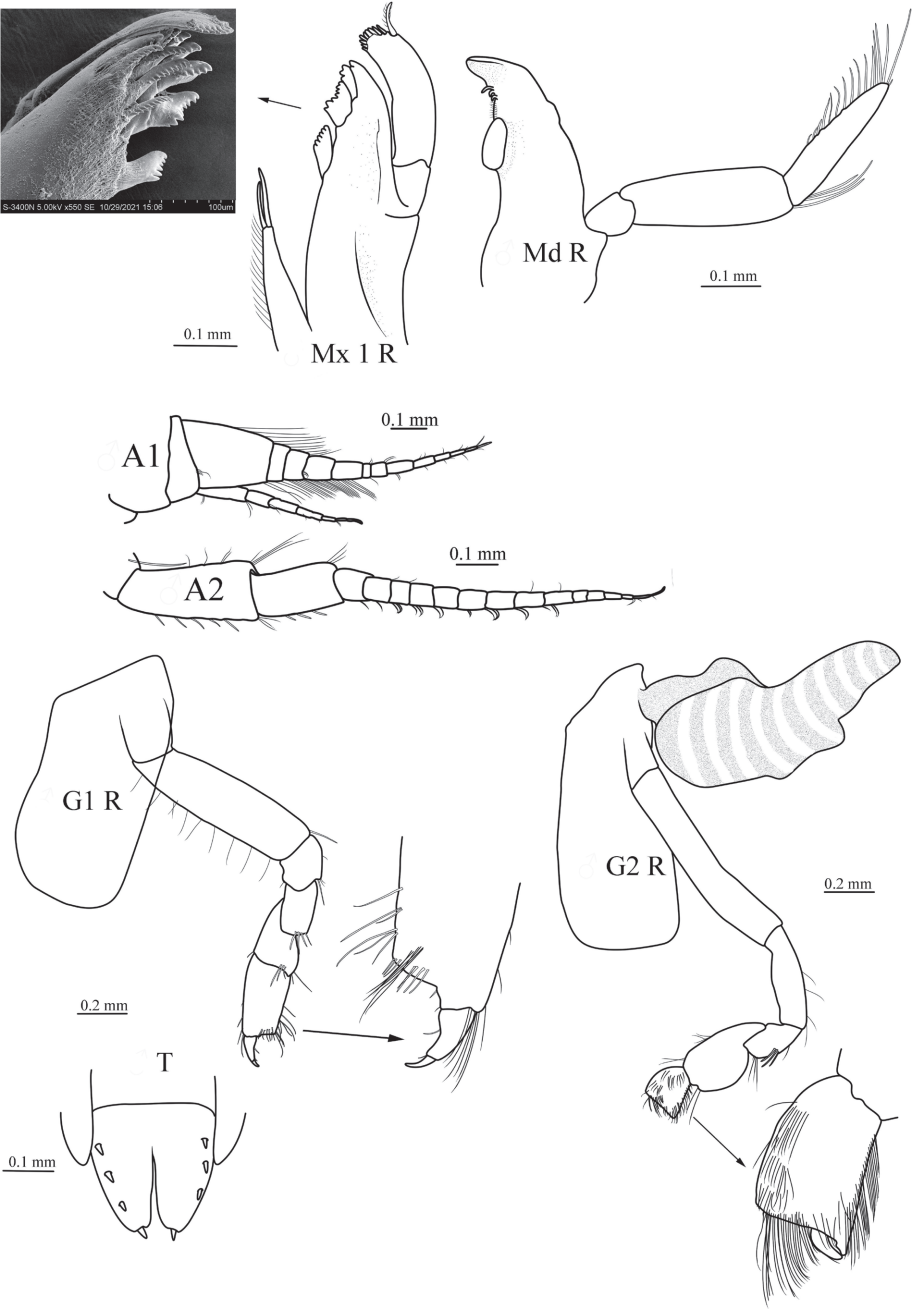
*Orchomenellacompressa* sp. nov., MBM 286555, paratype, ♂ (5.6 mm): Md R, right mandible; M×1 R, right maxilla 1 with SEM picture of outer plate; A1, antenna 1; A2, antenna 2; G1 R, right gnathopod; G2 R, right gnathopod 2.

**Figure 4.**
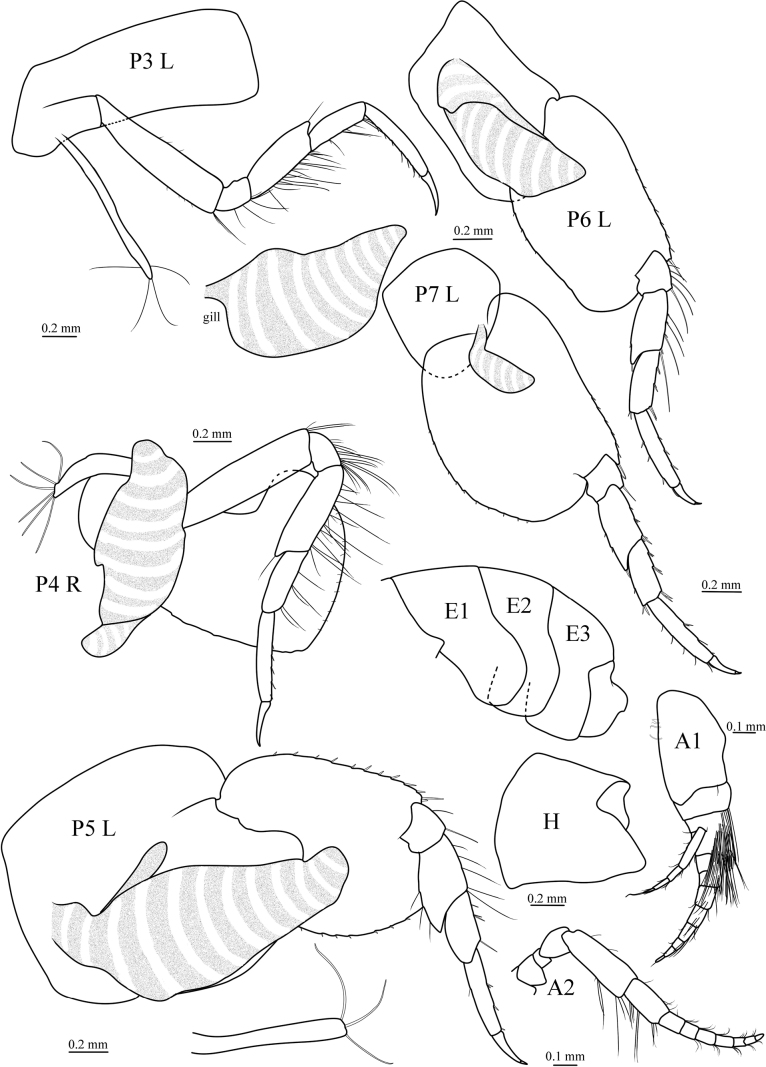
*Orchomenellacompressa* sp. nov., MBM 286555, holotype, ♀ (6.2 mm): P3 L, left pereopod 3; P4 L, left pereopod 4; P5 L, left pereopod 5; P6 L, left pereopod 6; P7 L, left pereopod 7; H, head; A1, antenna 1; A2, antenna 2; E1–3, epimeral plates 1–3.

#### Etymology.

From the Latin *compressa* (= compressed), referring to the compressed distal 3 articles of the gnathopod 2.

#### Distribution.

Presently known only from Okinawa Trough, at a depth of 1243 m.

#### Remarks.

**Figure 5.**
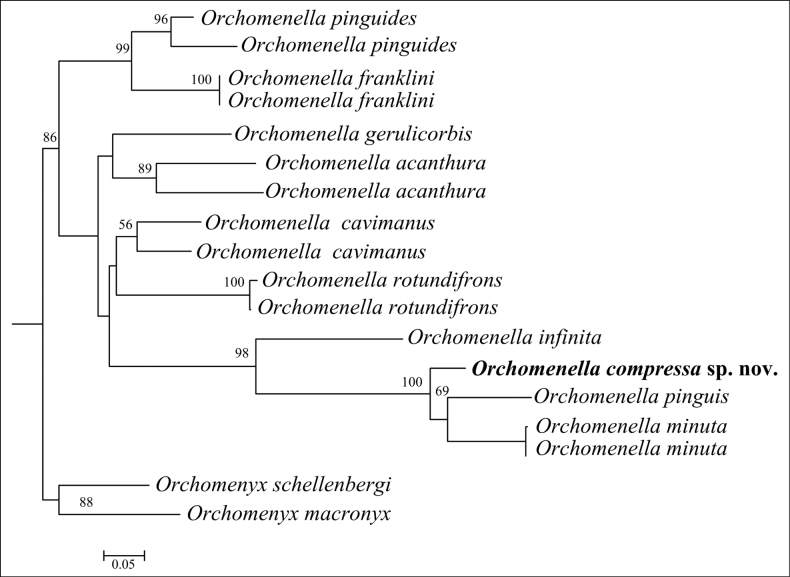
Phylogenetic relationships among *Orchomenellacompressa* sp. nov. and nine species of *Orchomenella* registered in GenBank, analyzed by the maximum-likelihood (ML) method with two *Orchomenyx* species as outgroup taxa. Bootstrap values of ML (>50) are indicated above branches of clades.

This is the first time that a species of *Orchomenella* has been reported from hydrothermal vents. The new species can be distinguished from other *Orchomenella* species in lacking of eyes and having the distal three articles of gnathopod 2 compressed. The new species is morphologically most similar to *O.tabasco* (Barnard, 1967), which was collected from the Cedros Trench at 1720–1728 m. However, *O.compressa* sp. nov. differs from *O.tabasco* by following characters: carpus of the gnathopod 1 shorter than propodus in the new species, rather than subequal in length in *O.tabasco*, and telson cleft more than 50% in the new species, rather than cleft only 40% in *O.tabasco* (Barnard 1967). The molecular analysis shows *O.compressa* sp. nov. clustering with *O.pinguis* (Boeck, 1861) and *O.minuta* (Krøyer, 1846) (Fig. 5). Morphologically, *O.compressa* sp. nov. can be distinguished from *O.pinguis* by the unserrated posterior margin of epimera 2 and 3 (Boeck 1861; Gurjanova 1962; Hirayama 1986), and from *O.minuta* by having three pairs of dorsal spines on the telson (Gurjanova 1962).

The ML tree inferred from partial COI sequences from 10 species of *Orchomenella*, including the new species, is shown in Fig. 5. *Orchomenellacompressa* sp. nov. is clustered with *O.pinguis* and *O.minuta* with high bootstrap support (100%). Interspecific genetic divergence (K2P) among these 10 species is summarized in Table 2. The pairwise distance was 0.09%–0.21%. The new species is closest to *O.pinguis* and *O.minuta* genetically (0.096 and 0.092, respectively), although morphological characters do not support this close relationship. It is a pity that the COI sequence of *O.tabasco*, which is morphologically most similar to *O.compressa* sp. nov., is still unavailable. Of the species analyzed, *O.rotundifrons* (Barnard, 1932), is most genetically divergent from the new species (0.215) (Havermans et al. 2010; Hupalo et al. 2022).

**Table 2.** Genetic divergence of the mitochondrial cytochrome c oxidase subunit I gene among the 10 species of *Orchomenella* calculated from Kimura 2-parameter corrected calculations.

	*O.compressa* sp. nov.	*O.pinguides*	*O.franklini*	*O.gerulicorbis*	*O.pinguis*	*O.minuta*	*O.cavimanus*	*O.rotundifrons*	*O.infinita*	*O.acanthura*
*O.compressa* sp. nov.	n/c									
*O.pinguides*	0.198	0.066								
*O.franklini*	0.198	0.110	0.000							
*O.gerulicorbis*	0.204	0.149	0.156	n/c						
*O.pinguis*	0.096	0.203	0.210	0.208	n/c					
*O.minuta*	0.092	0.205	0.206	0.204	0.109	0				
*O.cavimanus*	0.180	0.141	0.142	0.125	0.181	0.181				
*O.rotundifrons*	0.215	0.170	0.152	0.139	0.214	0.205	0.010			
*O.infinita*	0.164	0.190	0.178	0.176	0.190	0.170	0.186	n/c		
*O.acanthura*	0.190	0.177	0.167	0.158	0.210	0.198	0.169	0.192	0.134	n/c

## Supplementary Material

XML Treatment for Orchomenella compressa

## ﻿Additional information

### Author ORCIDs

Yanrong Wang https://orcid.org/0000-0002-7188-4232

Zhongli Sha https://orcid.org/0000-0002-2192-3758

### Data availability

All of the data that support the findings of this study are available in the main text or Supplementary Information.
